# The Impact of Water and Sanitation on Childhood Mortality in Nigeria: Evidence from Demographic and Health Surveys, 2003–2013

**DOI:** 10.3390/ijerph110909256

**Published:** 2014-09-05

**Authors:** Osita K. Ezeh, Kingsley E. Agho, Michael J. Dibley, John Hall, Andrew N. Page

**Affiliations:** 1 School of Medicine, University of Western Sydney, Locked Bag 1797, Penrith, NSW 2571, Australia; 2 School of Science and Health, University of Western Sydney, Locked Bag 1797, Penrith, NSW 2571, Australia; E-Mails: k.agho@uws.edu.au (K.A.); a.page@uws.edu.au (A.N.P.); 3 Sydney School of Public Health, Edward Ford Building (A27), University of Sydney, Sydney, NSW 2006, Australia; E-Mail: michael.dibley@sydney.edu.au; 4 School of Medicine and Public Health, Faculty of Health, University of Newcastle, Callaghan, NSW 2308, Australia; E-Mail: John.Hall@newcastle.edu.au

**Keywords:** mortality, water, sanitation, children, Nigeria

## Abstract

In Nigeria, approximately 109 million and 66 million people lack access to sanitation facilities and water, respectively. This study aimed to determine whether children under 5 years old without access to improved water and sanitation facilities are at higher risk of death in Nigeria. Pooled 2003, 2008 and 2013 Nigeria Demographic and Health Survey data were used to examine the impact of water and sanitation on deaths of children aged 0–28 days, 1–11 months, and 12–59 months using Cox regression analysis. Survival information of 63,844 children was obtained, which included 6285 deaths of children under 5 years old; there were 2254 cases of neonatal mortality (0–28 days), 1859 cases of post-neonatal mortality (1–11 months) and 2,172 cases of child mortality (1–4 years old). Over a 10-year period, the odds of neonatal, post-neonatal and child deaths significantly reduced by 31%, 41% and 47% respectively. The risk of mortality from both unimproved water and sanitation was significantly higher by 38% (Adjusted hazard ratios (HR) = 1.38, 95% confidence interval (CI): 1.14–1.66) for post-neonatal mortality and 24% (HR = 1.24, 95% CI: 1.04–1.48) for child mortality. The risk of neonatal mortality increased by 6% (HR = 1.06, 95% CI: 0.85–1.23) but showed no significant effect. The Nigerian government needs to invest more in water and sanitation to reduce preventable child deaths.

## 1. Introduction

Access to unimproved water and sanitation among children under 5 years old is a serious public health problem in many developing countries, including Nigeria [1]. Globally, nearly a billion people still lack access to improved sources of drinking water, and about 2.5 billion lack improved sanitation [2]. Unimproved water and sanitation are major causes of diarrhoea, which globally accounts for approximately 1.4 million child deaths each year. The majority of these deaths occur in sub-Saharan Africa where nearly half the population lacks access to improved water and sanitation [3]. Children are more vulnerable to the health hazards associated with unimproved water supply and sanitation; their immune, respiratory, and digestive systems are still developing [4], and children play in areas where contaminants may accumulate [5]. 

The impact of unimproved water and sanitation as a leading cause of childhood diarrhoea has long been recognized and documented in the public health literature. In response to this, in the past two decades the Nigerian government has launched and implemented the National Water Supply and Sanitation Policy, Presidential Water Initiative, and National Economic Empowerment and Development Strategy [6,7]. Despite all these initiatives, a recent report on global progress on sanitation and drinking water indicates that approximately 109 million and 66 million people in Nigeria still lack access to basic sanitation facilities and improved drinking water, respectively [2]. 

Past studies have shown that access to improved water and sanitation leads to a reduction in childhood mortality as well as child diarrhoea [8,9]. A recent large cross-sectional study undertaken in 38 developing countries concluded that access to improved water and sanitation can reduce child mortality by approximately 20%, and each year prevent about 2.2 million deaths in children aged under 5 years old from low-income and middle-income countries, excluding China [10]. 

The number of people with access to improved water and sanitation in Nigeria is very low, particularly in rural areas (48% for water and 28% for sanitation) [11], and could be one of the reasons why Nigeria still has the highest reported number of childhood deaths in Africa [12]. Each year in Nigeria, approximately 150,000 children under 5 years old, die from diarrhoea [13]. Therefore, a detailed understanding of the impact of water and sanitation on childhood mortality is needed to develop effective community mobilisation interventions aimed at reducing unimproved water and sanitation related deaths. Hence, this study aimed to use pooled data from the 2003, 2008 and 2013 Nigeria Demographic and Health Surveys (NDHS) to examine whether children under 5 years old without access to improved water sources and sanitation were at a greater risk of dying compared with children under 5 years old with access to improved water sources and sanitation. Specifically, we examined how well the combined effect of water and sanitation correlated with neonatal mortality (0–28 days), post-neonatal mortality (1–11 months), and child mortality (12–59 months). 

## 2. Methods

### 2.1. Data Source

Data for this study were obtained from NDHS, which are available online with ethics approval from ICF International (Calverton, MD, USA). NDHS have been conducted in Nigeria approximately every 5 years old since 1990 by the National Population Commission (NPC) in conjunction with ORC Macro international, and are largely sponsored by the United States Agency for International Development (USAID). The NDHS program collects data nationally on a wide range of socio-demographic and health characteristics such as childhood mortality, childhood illnesses, birth history, sex, education, and maternal and child health by interviewing women and men of reproductive age 15–49 and 15–59 years old, respectively. The information gathered from interviewees is recorded in three separate questionnaires. A multi-stage, stratified, cluster random sampling method was used to gather the information. This method has been discussed in detail elsewhere [11,14,15]. 

The data for the study were from the 2003, 2008 and 2013 NDHS. In this pooled dataset, information was available from 79,953 married women aged 15–49 years old, consisting of 7620 women from the 2003 survey, 33,385 women from the 2008 survey, and 38,948 women from the 2013 survey. The analyses used survival information from 63,844 singleton live-born infants of the most recent birth of a mother within five years prior to the mother’s interview [11,14,15]. Only each mother’s most recent birth was used in the analyses because only these births had detailed information on the use of perinatal health services, and to limit the potential for differential recall of events from mothers who had delivered at very different durations prior to the interview. Participation in NDHS has always been high with an average individual response rate of more than 94% for the surveys examined.

### 2.2. Outcome Variables

The main outcome variables in the study were neonatal mortality (death between birth and 28 days), post-neonatal mortality (death between 1 month and 11 months), and child mortality (death between 12 and 59 months). The outcome variables took a binary form such that child death was considered a “success” (1 = if death occurred in the specified age periods) or “failure” (0 = if child was alive in the specified age periods).

### 2.3. Exposure Variables

The key exposure variables examined were the main source of water and type of sanitation (or toilet) facilities available to members of the households. In the merged data set, there were 12 different categories of water source and 11 different categories of sanitation facility available to household members. In the analysis, these categories were classified as improved and unimproved according to WHO/UNICEF guidelines (Table 1).

In the analysis, water source and sanitation facilities were classified into four categories: improved water and improved sanitation, improved water and unimproved sanitation, unimproved water and improved sanitation, and unimproved water and unimproved sanitation facilities. The primary aim was to examine whether the combined impact of unimproved water and sanitation was associated with childhood mortality. 

**Table 1:** Classification of water sources and sanitation facilities based on WHO/UNICEF guidelines [2].

Variable	Improved	Unimproved
Water source	Piped water connection to household, public taps or standpipes, boreholes or tube wells, protected dug well, protected spring and rainwater collection.	Unprotected dug well, unprotected spring, cart with small tank or drum, surface water (e.g., river, dam, lake, pond, stream, canal or irrigation channel) and bottled water.
Sanitation facility	Pour-flush system, piped sewer system, septic tank, ventilated improved pit latrine (VIP) and pit latrine with slab.	Pit latrine without slab, bucket, hanging toilet or latrine, no facilities, bush or field and shared or public facility.

Note: N.B. sanitation refers to toilet facility.

### 2.4. Potential Confounding Variables

The confounding variables considered in the analyses were based on previous literature on water, sanitation, and child survival [4,10,16,17,18]. These confounding variables were classified into two distinct groups: *socioeconomic level factors*, and *demographic level factors*. The socioeconomic variables examined consisted of place of residence (urban or rural areas), mother’s education, and mother’s work status. Other socioeconomic variables included were *household wealth index*, *mother’s literacy level*, and *father’s level of education*. The three demographic level factors assessed were *mother’s age at child’s birth*, *perceived newborn size by the mother,* and *sex of child*. Perceived newborn size at birth by mothers *(small* or *very small*, and *average* or *large)* was used instead of birth weight at birth because more than half of the newborns were not weighed at birth. This measure was a reasonable proxy because a previous study showed that there is a close relationship between mean birth weight and perceived newborn size by the mother [19]. 

A household wealth index was constructed using a principal component analysis (PCA) [20]. Weights were assigned to the household facilities and assets of respondents. The assets included were those that were consistent across the pooled NDHS data; these were radio, television, fridge, bicycle, motorcycle, car, telephone, electricity, and type of floor material used in rooms. In the NDHS data set, the household wealth index was categorized into five quintiles: *poorest, poorer, middle, richer,* and *richest*. However, in the analysis, the household wealth index was re-categorised into three groups. The bottom 40% of households was arbitrarily referred to as poor households, the next 40% as middle households, and the top 20% as rich households.

### 2.5. Statistical Analysis

Initially, a frequency tabulation of all the potential confounding variables was conducted by the year of survey to describe the data used in the study, followed by estimation of the mortality rates by combined water sources and sanitation facilities, using a similar method to that described by Rutstien and Rojas [21]. To examine the impact of combined water sources and sanitation facilities, a multivariable analysis was conducted using a Cox proportional hazard regression model. 

For the multivariable model, a staged modelling technique was used. In the first stage, all the socioeconomic and demographic variables were entered into the baseline multivariable model to assess their relationship with the study outcomes. A stepwise backwards elimination process was conducted and variables that were significantly associated with the study outcomes at a 5% significance level were retained in the model (*model 1*). 

In the second stage, *water source* was independently investigated with the socioeconomic and demographic variables that were significantly associated with the mortality outcomes, and those variables with *p*-values < 0.05 were retained (*model 2*). In the third stage, *sanitation facility* was independently examined with the socioeconomic and demographic variables that were significantly associated with the mortality outcomes. As before, those variables with *p*-values <0.05 were retained (*model 3*). In the final stage, a similar procedure was used for the combined exposure variables (water and sanitation), which were entered into *model 1*, and those variables with *p*-values <0.05 were retained in the final model (*model 4*). 

The hazard ratios (HR) and their 95% confidence intervals obtained from the adjusted Cox proportional Hazard models were used to measure the impact of the combined effect of water sources and sanitation facilities on neonatal, post-neonatal, and child mortality. All analyses were conducted using “SVY” commands in STATA version 12.0 (Stata Corporation, College Station, TX, USA) to adjust for the cluster sampling survey design, weights, and calculate standard errors.

## 3. Results and Discussion

A weighted total of 63,844 most recent live births of children younger than 5 years old occurred within the 5 year period prior to the survey interview date, including 6285 deaths. Of these deaths, 2254 occurred between birth and 28 days (neonatal mortality), 1859 between 1 and 11 months (post-neonatal mortality) and 2172 between 1 and 4 years old (child mortality). Table 2 shows the distribution of the exposure and confounding variables by year of survey. Place of residence was nearly equally represented in the three study periods. The proportion of children whose mothers had secondary or higher education slightly increased from 24.5% in 2003 to 31.5% in 2013. Both male and female children were nearly equally represented in the three study periods, while the proportion of children whose mothers were from poor households decreased from 42.3% in 2003 to 33.6% in 2013. In 2003, approximately 60% of children under 5 years old lived in households having both unimproved water and sanitation, and this decreased to 27.8% in 2008, and was 27.5% in 2013. 

The neonatal mortality rate (NMR) was found to be higher among newborns born to mothers in households with access to both unimproved water sources and unimproved sanitation facilities (NMR: 38.2 *vs.* 32.6). The post-neonatal mortality rate (PMR) in households with access to unimproved water sources and sanitation was greater than those with access to improved water and sanitation (PMR: 35.7 *vs.* 20.2). Similarly, the child mortality rate (CMR) for children aged 1–4 years old was higher in households with no access to both improved water sources and sanitation facilities (CMR: 40.8 *vs.* 23.9).

**Table 2:** The prevalence of singleton live birth of infants under 5 years old as reported by mothers interviewed during demographic and health surveys in Nigeria, 2003–2013 (n = 63,844).

Variable	Total Live Births		% of Weighted Total ˟	Prevalence ˟ by Survey (%)		
	Unweighted ʶ	Weighted ˟		2003	2008	2013
***Household environmental factor***						
**Water (n = 61,995)**						
Improved water	31,530	32,821	51.4	35.4	50.4	55.4
Unimproved water	30,531	29,174	45.7	62.9	47.7	40.6
**Sanitation (n = 62,994)**						
Improved	28,416	30,086	47.1	12.6	51.1	50.3
Unimproved	34,511	32,908	51.6	85.8	47.0	48.9
**Combined water and sanitation (n = 61,648)**						
Improved water, improved sanitation	18,106	19,680	30.8	9.7	31.2	34.6
Improved water, unimproved sanitation	13,299	13,013	20.4	25.7	18.8	20.7
Unimproved water, improved sanitation	9420	9370	14.7	2.9	19.1	13.1
Unimproved water, unimproved sanitation	20,878	19,585	30.7	60.0	27.8	27.5
***Socioeconomic factor***						
**Residence type**						
Urban	19,305	20,478	32.1	28.8	29.7	34.8
Rural	44,553	43,366	67.9	71.2	70.3	65.2
***Household wealth index***						
Poor	25,243	23,783	37.3	42.3	40.2	33.6
Middle	25,262	25,569	40.1	40.5	37.7	42.0
Rich	13,353	14,492	22.7	17.2	22.1	24.3
**Mother’s education **						
No education	31,203	30,947	48.5	51.8	46.7	49.4
Primary	13,871	13,539	21.2	23.6	23.0	19.1
Secondary or higher	18,784	19,357	30.3	24.5	30.2	31.5
**Mother’s literacy level (n = 63,532)**						
Cannot read at all	38,905	38,283	60.0	63.9	59.4	59.7
Able to read	24,668	25,249	39.6	35.8	40.1	39.8
**Father’s education (n = 61,964)**						
No education	24,634	24,365	38.2	39.7	37.1	38.8
Primary	12,825	12,843	20.1	23.0	21.5	18.4
Secondary or higher	24,431	24,756	38.8	34.0	38.2	40.2
**Mother’s working status (n = 61,857)**						
Not working	21,544	21,289	33.3	37.4	34.6	31.5
Working	40,328	40,568	63.5	62.6	63.1	64.1
***Demographic factor***						
**Currently breastfeeding**						
Yes	35,885	35,691	55.9	58.2	56.4	55.0
No	27,973	28,153	44.1	41.8	43.6	45.0
**Mother’s age ( years)**						
<20	3460	3473	5.4	7.3	5.4	5.1
20–29	30,822	30,870	48.4	51.6	48.2	47.8
30–39	23,035	23,136	36.2	32.6	36.1	37.1
40–49	6541	6366	10.0	8.6	10.3	10.0
**Sex of child**						
Female	31,385	31,491	49.3	48.8	49.1	49.6
Male	32,473	32,353	50.7	51.2	50.9	50.4
**Mother’s perceived baby size (n = 62,563)**						
Small or very small	9107	9023	14.1	14.2	13.8	14.4
Average or larger	53,448	53,540	83.9	84.5	83.9	83.7

Notes: **ʶ** The total unweighted number varies between categories because of some missing values. **˟** Weighting was applied to compensate for the multistage sampling design.

### 3.1. The Combined Effect of Water and Sanitation on Neonatal-Mortality

**Figure 1:**
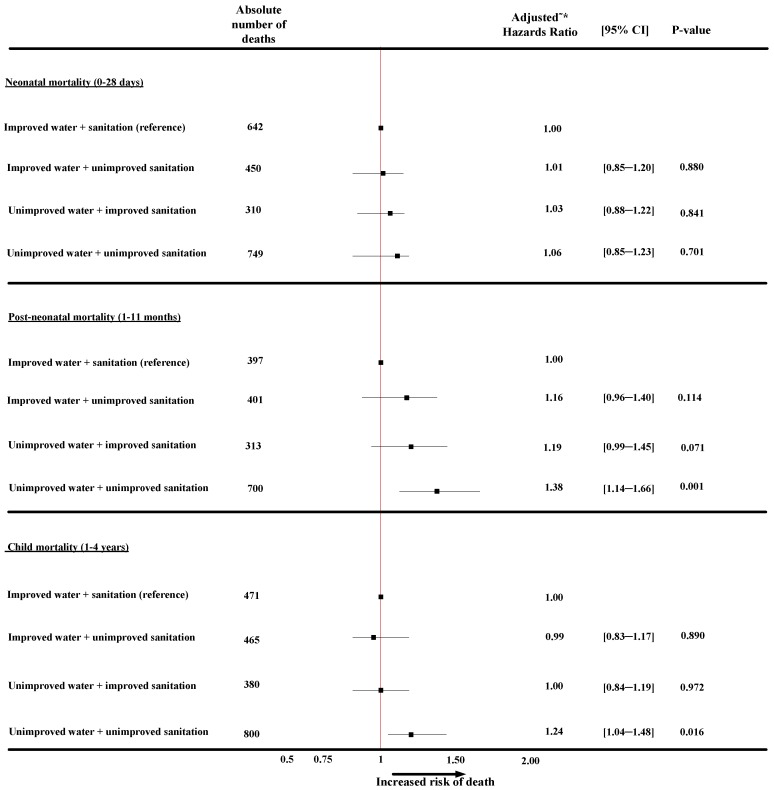
The combined effect of water and sanitation on neonatal, post-neonatal and child mortality in Nigeria, 2003–2013.

Notes: * Independent variables adjusted for were: place of residence, wealth index, mother’s (education, literacy level, working status, age), father’s education, child’s gender, currently breastfeeding and baby size.

Figure 1 presents findings from the multivariate analyses of the combined effect of water and sanitation on neonatal, post-neonatal, and child mortality after adjusting for confounding factors. The results show that neonates born to mothers in households with access to both unimproved water and sanitation had a higher risk of neonatal death (HR = 1.06; CI: 0.85―1.23) compared with the reference category (improved water and improved sanitation), though it was not statistically significantly different.

Table 3 (*model 4*) shows other significant factors that affected neonatal deaths in addition to unimproved water and sanitation including neonates born to mothers under 20 years old (HR = 3.45; CI: 2.79–4.27), newborns whose body size was perceived by their mothers as small or smaller (HR = 1.93; CI: 1.70–2.20), male newborns (HR = 1.38; CI: 1.24–1.55), newborns from poor households (HR = 1.36; CI: 1.12–1.65), and newborns not currently breastfed were 1.95 times at higher risk of neonatal death (HR = 1.95; CI: 1.73–2.20). In the final model, we removed the household wealth index and replaced it with place of residence. In addition to the impact of water and sanitation, newborns born to mothers residing in rural areas had a significantly higher risk of neonatal death (HR = 1.35; CI: 1.17–1.57) than those newborns born in urban areas. 

### 3.2. The Combined Effect of Water and Sanitation on Post-Neonatal Mortality

Compared with the reference category in Figure 1, households with an unimproved source of water and unimproved sanitation (HR = 1.38; CI: 1.14–1.66) reported a significantly higher risk of post-neonatal mortality. Table 4 (*model 4*) shows other factors that significantly affected post-neonatal deaths in addition to households having both unimproved source of water and unimproved sanitation. Infants whose fathers had no formal education were more likely to die (HR = 1.22; CI: 1.05–1.42), as were infants whose birth size was perceived as small or smaller (HR = 1.18; CI: 1.03–1.36). Infants that were not currently breastfed had a significantly higher risk of post-neonatal mortality (HR = 1.52; CI: 1.34–1.71) compared with currently breastfed infants. 

**˜** Missing values were not included.

**Table 3:** Model for neonatal mortality.

Variables	(Model 0) ˇ	(Model 1) ^	(Model 2) ^	(Model 3) ^	(Model 4) ^
	HR (95% CI)	HR (95% CI)	HR (95% CI)	HR (95% CI)	HR (95% CI)
**Year of survey**					
2003	*Ref*	*Ref*	*Ref*	*Ref*	*Ref*
2008	0.83 (0.69–1.01)	0.86 (0.71–1.03)	0.86 (0.71–1.03)	0.86 (0.71–1.04)	0.86 (0.71–1.04)
2013	0.68 (0.56–0.82)	0.69 (0.57–0.83)	0.69 (0.57–0.84)	0.69 (0.57–0.84)	0.69 (0.57–0.85)
***Socioeconomic factor***					
**Residence type**					
Urban	*Ref*				
Rural	1.46 (1.27–1.67)				
**Household wealth index**					
Rich	*Ref*				
Poor	1.51 (1.28–1.78)	1.38 (1.16–1.64)	1.37 (1.14–1.64)	1.37 (1.13–1.65)	1.36 (1.12–1.65)
Middle	1.28 (1.08–1.52)	1.23 (1.04–1.46)	1.22 (1.03–1.46)	1.22 (1.02–1.46)	1.22 (1.02–1.46)
**Mother’s education**					
Secondary or higher	*Ref*				
No education	1.24 (1.07–1.44)				
Primary	1.24 (1.06–1.46)				
**Mother’s literacy level**					
Able to read	*Ref*				
Cannot read at all	1.24 (1.10–1.40)				
**Father’s education**					
Secondary or higher	*Ref*				
No education	1.15 (1.02–1.31)				
Primary	1.14 (0.98–1.32)				
**Mother’s working status**					
Not working	*Ref*				
Working	0.83 (0.75–0.94)				
***Demographic factor***					
**Mother’s age**					
30–39	*Ref*	*Ref*		*Ref*	*Ref*
<20	3.69 (2.98–4.58)	3.46 (2.79–4.28)	3.45 (2.79–4.27)	3.46 (2.79–4.28)	3.45 (2.79–4.27)
20–29	1.17 (1.03–1.32)	1.17 (1.03–1.33)	1.17 (1.03–1.33)	1.17 (1.04–1.33)	1.17 (1.03–1.33)
40–49	1.21 (1.00–1.47)	1.09 (0.90–1.33)	1.09 (0.90–1.33)	1.09 (0.90–1.33)	1.09 (0.90–1.33)
**Sex of child**					
Female	*Ref*	*Ref*	*Ref*	*Ref*	*Ref*
Male	1.37 (1.23–1.53)	1.39 (1.24–1.55)	1.39 (1.24–1.55)	1.39 (1.24–1.55)	1.38 (1.24–1.55)
**Mother’s perceived baby size**					
Average or larger	*Ref*	*Ref*	*Ref*	*Ref*	*Ref*
Small or very small	1.97 (1.74–2.34)	1.93 (1.70–2.20)	1.93 (1.70–2.20)	1.94 (1.70–2.20)	1.93 (1.70–2.20)
**Currently breastfeeding**					
Yes	*Ref*	*Ref*	*Ref*	*Ref*	*Ref*
No	1.83 (1.62–2.06)	1.95 (1.73–2.20)	1.95 (1.73–2.20)	1.95 (1.73–2.20)	1.95 (1.73–2.20)
***Household environmental factors***					
**Source of water**					
Improved	*Ref*		*Ref*		
Unimproved	1.18 (1.06–1.33)		1.02 (0.91–1.15)		
**Type of sanitation facility**					
Improved	*Ref*			*Ref*	
Unimproved	1.20 (1.07–1.35)			1.02 (0.89–1.16)	

Notes: **^** Independent variables adjusted were: year of survey, place of residence, wealth index, mother’s (education, literacy level, working-status, age), father’s education, child’s gender, currently breastfeeding and baby size; **ˇ** Model 0—unadjusted independent variables; Model 1—Independent variables associated with child mortality; Model 2—Model 1 plus water; Model 3—Model 1 plus sanitation; Model 4—Model 1 plus combined water and sanitation.

As shown in Table 4 (*model 4*), there was a significantly higher risk of post-neonatal death for infants born to mothers from poor households (HR = 1.60; CI: 1.27–2.03) and middle-class households (HR = 1.46; CI: 1.18–1.80) compared with infants from rich households. Infants born to mothers under 20 years old had a 3.07 times greater risk of dying than those born to mothers aged 20 years old or more (HR = 3.07; CI: 2.42–3.90). 

**Table 4:** Model for post-neonatal mortality.

Variables	(Model 0) ˇ	(Model 1) ^	(Model 2) ^	(Model 3) ^	(Model 4) ^
	HR (95% CI)	HR (95% CI)	HR (95% CI)	HR (95% CI)	HR (95% CI)
**Year of survey**					
2003	*Ref*	*Ref*	*Ref*	*Ref*	*Ref*
2008	0.70 (0.58–0.85)	0.73 (0.61–0.89)	0.75 (0.62–0.91)	0.74 (0.60–0.90)	0.73 (0.60–0.90)
2013	0.55 (0.45–0.67)	0.57 (0.47–0.70)	0.59 (0.48–0.73)	0.58 (0.47–0.71)	0.59 (0.47–0.72)
***Socioeconomic factor***					
**Residence type**					
Urban	*Ref*				
Rural	1.51 (1.30–1.75)				
**Household wealth index**					
Rich	*Ref*	*Ref*	*Ref*	*Ref*	*Ref*
Poor	2.08 (1.72–2.51)	1.75 (1.42–2.15)	1.64 (1.32–2.04)	1.73 (1.37–2.18)	1.60 (1.27–2.03)
Middle	1.72 (1.42–2.07)	1.56 (1.28–1.89)	1.51 (1.23–1.84)	1.55 (1.26–1.89)	1.46 (1.18–1.80)
**Mother’s education**					
Secondary or higher	*Ref*				
No education	1.57 (1.34–1.83)				
Primary	1.21 (1.01–1.45)				
**Mother’s literacy level**					
Able to read	*Ref*				
Cannot read at all	1.54 (1.35–1.76)				
**Father’s education**					
Secondary or higher	*Ref*	*Ref*	*Ref*	*Ref*	*Ref*
No education	1.53 (1.33–1.75)	1.24 (1.07–1.45)	1.23 (1.06–1.43)	1.25 (1.07–1.45)	1.22 (1.05–1.42)
Primary	1.32 (1.11–1.57)	1.15 (0.97–1.37)	1.15 (0.96–1.36)	1.15 (0.97–1.37)	1.14 (0.96–1.36)
**Mother’s working status**					
Not working	*Ref*				
Working	0.86 (0.77–0.96)				
***Demographic factor***					
**Mother’s age**					
30–39	*Ref*	*Ref*	*Ref*	*Ref*	*Ref*
< 20	3.54 (2.80–4.47)	3.10 (2.44–3.95)	3.08 (2.42–3.91)	3.10 (2.44–3.95)	3.07 (2.42–3.90)
20–29	1.18 (1.04–1.33)	1.15 (1.02–1.31)	1.15 (1.02–1.31)	1.15 (1.02–1.31)	1.15 (1.02–1.31)
40–49	1.01 (0.83–1.23)	0.89 (0.74–1.09)	0.90 (0.74–1.09)	0.89 (0.74–1.09)	0.90 (0.74–1.09)
**Sex of child**					
Female	*Ref*				
Male	1.03 (0.92–1.15)				
**Mother’s perceived baby size**					
Average or larger	*Ref*	*Ref*	*Ref*	*Ref*	*Ref*
Small or very small	1.29 (1.12–1.49)	1.19 (1.03–1.37)	1.18 (1.03–1.36)	1.17 (1.02–1.35)	1.18 (1.03–1.36)
**Currently breastfeeding**					
Yes	*Ref*	*Ref*	*Ref*	*Ref*	*Ref*
No	1.37 (1.22–1.55)	1.51 (1.34–1.70)	1.51 (1.34–1.71)	1.51 (1.34–1.70)	1.52 (1.34–1.71)
***Household environmental factors***					
**Source of water**					
Improved	*Ref*		*Ref*		
Unimproved	1.44 (1.27–1.62)		1.16 (1.02–1.31)		
**Type of sanitation facility**					
Improved	*Ref*			*Ref*	
Unimproved	1.35 (1.19–1.53)			1.02(0.88–1.17)	

Notes: **^** Independent variables adjusted were: place of residence, wealth index, mother’s (education, literacy level, working status, age), father’s education, child’s gender, currently breastfeeding and baby size; **ˇ** Model 0—unadjusted independent variables; Model 1—Independent variables associated with child mortality; Model 2—Model 1 plus water; Model 3—Model 1 plus sanitation; Model 4—Model 1 plus combined water and sanitation.

### 3.3. The Combined Effect of Water and Sanitation on Child Mortality

As shown in Figure 1, children aged between 1 and 4 years old living in households with access to both an unimproved source of water and sanitation facilities had a greater risk of child mortality (HR = 1.24; CI: 1.04―1.48) compared with the reference category. Table 5 (*model 4*), shows other factors associated with a significantly higher risk of child mortality in addition to unimproved water and unimproved sanitation. 

**Table 5:** Model for child mortality.

Variables	(Model 0) ˇ	(Model 1) ^	(Model 2) ^	(Model 3) ^	(Model 4) ^
	HR (95% CI)	HR (95% CI)	HR (95% CI)	HR (95% CI)	HR (95% CI)
**Year of survey**					
2003	*Ref*	*Ref*	*Ref*	*Ref*	*Ref*
2008	0.76 (0.62–0.93)	0.79 (0.66–0.95)	0.80 (0.67–0.97)	0.76 (0.63–0.92)	0.76 (0.63–0.92)
2013	0.52 (0.42–0.63)	0.54 (0.45–0.66)	0.55 (0.45–0.67)	0.52 (0.43–0.64)	0.53 (0.43–0.64)
***Socioeconomic factor***					
**Residence type**					
Urban	*Ref*				
Rural	2.31 (1.98–2.69)				
**Household wealth index**					
Rich	*Ref*	*Ref*	*Ref*	*Ref*	*Ref*
Poor	3.21 (2.68–3.84)	2.29 (1.87–2.79)	2.21 (1.80–2.71)	2.45 (1.97–3.06)	2.33 (1.86–2.90)
Middle	2.37 (1.98–2.84)	1.95 (1.61–2.36)	1.91 (1.58–2.32)	2.03 (1.67–2.47)	1.95 (1.60–2.37)
**Mother’s education**					
Secondary or higher	*Ref*				
No education	3.13 (2.64–3.72)				
Primary	2.04 (1.68–2.47)				
**Mother’s literacy level**					
Able to read	*Ref*				
Cannot read at all	2.46 (2.14–2.84)				
**Father’s education**					
Secondary or higher	*Ref*	*Ref*	*Ref*	*Ref*	*Ref*
No education	2.36 (2.05–2.71)	1.80 (1.54–2.09)	1.78 (1.53–2.08)	1.79 (1.53–2.08)	1.76 (1.51―2.06)
Primary	1.78 (1.53–2.07)	1.45 (1.24–1.69)	1.44 (1.24–1.68)	1.45 (1.24–1.69)	1.44 (1.23―1.68)
**Mother’s working status**					
Not working	*Ref*				
Working	0.81 (0.72–0.91)				
***Demographic factor***					
**Mother’s age**					
30–39	*Ref*				
<20	1.54 (1.15–2.05)				
20–29	1.10 (0.98–1.22)				
40–49	1.20 (1.03–1.41)				
**Sex of child**					
Female	*Ref*				
Male	1.05 (0.95–1.17)				
**Mother’s perceived baby size**					
Average or larger	*Ref*				
Small or very small	1.35 (1.17–1.55)				
**Currently breastfeeding**					
Yes	*Ref*	*Ref*	*Ref*	*Ref*	*Ref*
No	1.03 (0.93–1.14)	1.13 (1.02–1.25)	1.13 (1.02–1.26)	1.13 (1.02–1.25)	1.13 (1.02―1.26)
***Household environmental factors***					
**Source of water**					
Improved	*Ref*		*Ref*		
Unimproved	1.50 (1.33–1.69)		1.09 (0.96–1.23)		
**Type of sanitation facility**					
Improved	*Ref*			*Ref*	
Unimproved	1.34 (1.19–1.51)			0.89 (0.79–1.02)	

Notes: **^** Independent variables adjusted were: place of residence, wealth index, mother’s (education, literacy level, working status, age), father’s education, child’s gender, currently breastfeeding and baby size; **ˇ** Model 0—unadjusted independent variables; Model 1—Independent variables associated with child mortality; Model 2—Model 1 plus water; Model 3—Model 1 plus sanitation; Model 4—Model 1 plus combined water and sanitation.

These include children that were not currently breastfed (HR = 1.13; CI: 1.02–1.26), children whose fathers had no formal education (HR = 1.76; CI: 1.51–2.06), and children from poor households (HR = 2.33; CI: 1.86–2.90). 

### 3.4. Discussion

In this study, the impact of water and sanitation on neonatal, post-neonatal, and child mortality was examined. We found that unimproved water and sanitation significantly increased the risk of post-neonatal and child mortality; however, it had no significant effect on the risk of neonatal mortality. This pattern is consistent with previous studies conducted in Egypt and Eritrea [17,22]; these authors reported that the impact of household environmental factors is very weak during the neonatal period; however, there was a large and statistically significant impact during the post-neonatal and child periods. A possible explanation of our findings is that exclusive breastfeeding has important protective effects on the survival of infants, increases immunity, and decreases the risk of prolonged diarrhoea; neonates are less likely to be exposed to pathogens in contaminated water [23,24,25,26]. The large impact of breastfeeding observed during the neonatal and post-neonatal periods reaffirms the protective effects of breastfeeding in reducing the risk of infant mortality. 

The effect of unimproved water and sanitation was substantial during the post-neonatal period. Children learn to crawl and walk during this period, and they experience increased exposure to pathogens that cause diarrhoea from a variety of environmental sources, including contaminated water [5]. It is also during this period that the weaning process commences, and low income households often use unimproved water to prepare weaning foods, thereby transmitting pathogens to infants that cause diarrheal diseases, which has been shown to result in high mortality [27,28,29]. Similarly, children aged between 1 and 4 years old exposed to unimproved water and sanitation had increased mortality risk, though the effect was lower compared with that of the post-neonatal period. The lower mortality risk reported among children aged between 1 and 4 years old could be attributed to their relatively well developed immune response to pathogens compared with that during the infancy period; for example, maternal immunoglobulin is weak, short lived, and of low avidity during the first year of life [30]. 

Findings from this study showed that household economic status influenced survival of children under 5 years old. During all age periods, children from poor households had a significantly higher risk of mortality compared with children from rich households [17,31]. This finding is not surprising because wealthier households were more likely to have improved water sources and excreta disposal facilities than poor households. There was a strong effect in the final model when we replaced the household wealth index with place of residence, indicating that household dwelling had a significant influence on neonatal, post-neonatal, and child mortality. This finding is supported by other studies [32,33] that have found rural dwellings to be a strong predictor of mortality of children under 5 years old. Limited access to health facilities and maternal healthcare services disproportionally hinders rural dwellers from receiving adequate healthcare services, resulting in a high probability of child death. In Nigeria, as in many low and medium income countries, the majority of well-equipped hospitals and health centres are located in urban areas. 

We found that male neonates had a significantly higher risk of dying during the neonatal period compared with female neonates. This finding is consistent with a cross-sectional study conducted in Kenya in 2007, which reported that male neonates were 1.34 times at greater risk of dying than female neonates [34]. Biological factors are a possible explanation for this disparity [35]. The findings further indicated that maternal age at birth (<20 years old) and perception of newborn size at birth (small or very small) by mothers were strongly related to both neonatal and post-neonatal mortality, though they were not significantly related to child mortality. Paternal education had a strong impact on post-neonatal and child mortality. Children whose fathers had a secondary education or higher had significantly lower risk of death than those whose fathers had a primary or no formal education. This finding is consistent with studies in Bangladesh and Pakistan [31,36] which showed a strong relationship between childhood mortality and the level of education of the father. The lower risk of death observed for children whose fathers were educated may be because educated fathers are more likely to invest in both improved water sources and sanitation facilities [16]. 

The definitions of indicators (*water and sanitation*) used in this study were based on those recommended by WHO; other important strengths of the study were restricting analyses to the most recent births within 5 years prior to each survey to minimise any recall bias on birth and death dates reported by mothers, using a nationally representative survey with appropriate adjustment for sampling design and sampling weight, and the high response rate (95%) to the survey. However, limitations to be considered when interpreting the results include that no firm conclusions can be made on causes and effects in a cross-sectional design, and that other factors such as water storage facilities, quality and safety of the water sources as well as information on maintenance of existing water supply infrastructure [37] were not considered. Another possible limitation of this study is that information on medical status of children under 5 years old and causes of death were lacking in the NDHS data.4. 

## 4. Conclusions

Our analyses of the impact of water and sanitation in Nigeria indicated that children under 5 years old in households with access to both unimproved water sources and sanitation facilities had increased risk of neonatal, post-neonatal, and child death than children with access to improved water sources and sanitation. In addition to the effect of unimproved water and sanitation, the results revealed that households in rural areas, poor households, mother’s age at birth (<20 years old), mothers who perceived their newborns to be smaller than average at birth, and illiterate parents had a significantly higher risk of neonatal, post-neonatal, and child mortality. The findings from the study showed that Water and Sanitation community-based interventions are needed to prevent child deaths, and that such interventions should target low socioeconomic households in Nigeria.
